# Insectivorous bat activity dataset across different land-use types in the Islands of São Tomé and Príncipe, Central West Africa

**DOI:** 10.3897/BDJ.12.e131955

**Published:** 2024-09-05

**Authors:** Ana Filipa Palmeirim, Ana Catarina Araújo-Fernandes, Ana Sofia Castro-Fernandes, Patricia Guedes, José Cassari, Vanessa A. Mata, Natalie Yoh, Ricardo Rocha, Javier Martínez-Arribas, Fernanda Alves-Martins

**Affiliations:** 1 CIBIO, Centro de Investigação em Biodiversidade e Recursos Genéticos, InBIO Laboratório Associado, Campus de Vairão, Universidade do Porto, Vairão, Portugal CIBIO, Centro de Investigação em Biodiversidade e Recursos Genéticos, InBIO Laboratório Associado, Campus de Vairão, Universidade do Porto Vairão Portugal; 2 BIOPOLIS Program in Genomics, Biodiversity and Land Planning, CIBIO, Campus de Vairão, Vairão, Portugal BIOPOLIS Program in Genomics, Biodiversity and Land Planning, CIBIO, Campus de Vairão Vairão Portugal; 3 EcoHealth Alliance, New York City, United States of America EcoHealth Alliance New York City United States of America; 4 Monte Pico Association, Monte Café, São Tomé and Príncipe Monte Pico Association Monte Café São Tomé and Príncipe; 5 Durrell Institute of Conservation and Ecology (DICE), School of Anthropology and Conservation, Canterbury, United Kingdom Durrell Institute of Conservation and Ecology (DICE), School of Anthropology and Conservation Canterbury United Kingdom; 6 Department of Biology, University of Oxford, Oxford, United Kingdom Department of Biology, University of Oxford Oxford United Kingdom

**Keywords:** Chiroptera, Gulf of Guinea, habitat modification, tropical forests, oceanic islands, passive acoustic monitoring

## Abstract

**Background:**

São Tomé and Príncipe oceanic islands, in Central West Africa, are characterised by exceptional levels of endemism. Since human colonisation in the mid-15^th^ century, São Tomé and Príncipe have lost 74% and 67% of their native habitat, respectively. Today, these islands are mainly covered by remaining old-growth forests, secondary regrowth forests, shaded plantations (mostly of cocoa), oil palm plantations (in the case of São Tomé), small-scale agricultural areas and urban areas. Yet, little is known about how species on these islands are coping with land-use changes. Island ecosystems are particularly important for bats, with about 25% of the world’s bat species being entirely restricted to island systems. São Tomé and Príncipe Islands comprise six and four native insectivorous bats, respectively. Two species, *Chaerephontomensis* and *Macronycteristhomensis*, are island-endemics in São Tomé; *Pseudoromiciaprincipis* is an island-endemic in Príncipe; and *Miniopterusnewtoni* is endemic from both São Tomé and Príncipe. Here, we present a dataset comprising a comprehensive compilation of occurrence records derived from acoustic sampling of insectivorous bats across the predominant land-use types of both the São Tomé and Príncipe Islands. In each sampling site, standardised surveys consisted of deploying one Audio Moth device that recorded for 1 minute every 5 minutes over a 48-hour period. We identified a total of 19,437 bat-passes across the 115 sites surveyed in São Tomé Island and 17,837 bat-passes across the 50 sites surveyed in Príncipe Island.

**New information:**

Based on a sampling effort of 1,584 hours of recordings manually processed to identify all the contained bat passes, this dataset, publicly available on GBIF, provides comprehensive information on the activity of insectivorous bats across two endemic-rich oceanic islands in the Gulf of Guinea. For each bat pass identified, we report the identified species, geographic coordinates, land-use type, altitude, date and time. This is the first public dataset providing detailed information on species-level habitat use for insectivorous bats on oceanic islands in Africa.

## Introduction

Oceanic islands comprise important areas for biodiversity conservation, often harbouring a greater number of endemic species compared to equivalent mainland areas (Fernández-Palacios et al. 2021). Yet, islands have been significantly impacted by historical and contemporary human-induced changes in land-use and their relatively smaller size places them in a more vulnerable position to habitat disturbance (Norder et al. 2020, Nogué et al. 2021). Indeed, islands typically harbour high numbers of threatened species, have experienced more species extinctions and surviving taxa face particularly high rates of extinction debt (Triantis et al. 2010, Leclerc et al. 2018). Insular endemic species tend to be particularly vulnerable as their often higher habitat specialisation and unique adaptations, in addition to their interspecific interactions, aggravate the impacts of land-use change and other anthropogenic stressors, such as invasive species and climate change (Fernández-Palacios et al. 2021).

Island ecosystems are particularly important for bats, with ca. 25% of global bat species being entirely restricted to island systems (Jones et al. 2010). Due to their ability to fly, they can disperse over water and are often the only native mammals in many insular ecosystems (Fleming and Racey 2010). Bats play a crucial role in ecosystem functioning, providing valuable ecosystem services, such as pest control, facilitating seed dispersal and promoting pollination (Williams-Guillén and Perfecto 2011, Florens et al. 2017, Kemp et al. 2019). Despite their ecological significance, it is noteworthy that island-endemic bat populations are substantially more threatened than their non-insular counterparts (Conenna et al. 2017). Yet, island bats are poorly studied, which precludes efficient evidence-based guidelines for improving their conservation.

Located off the western equatorial coast of Central Africa, São Tomé and Príncipe Islands are part of the three oceanic islands that arose as part of the Cameroon Volcanic Line comprising the Gulf of Guinea (Ceríaco et al. 2022). Despite their relatively short distance from mainland Africa (250 and 220 km, respectively), these islands remained isolated throughout their geological history. As a result, São Tomé and Príncipe Islands are characterised by exceptional levels of endemism. For instance, of the eight insectivorous bat species these islands host, four of them are island-endemics (Rainho et al. 2022). São Tomé is home to the molossid *Chaerephontomensis* (Juste & Ibañez, 1993) and hipposiderid *Macronycteristhomensis* (Bocage, 1891). While the second is widespread across São Tomé, little is known about the former endemic species (Rainho et al. 2010, Rainho et al. 2022). Príncipe Island is home to a recently described endemic, the vespertilionid *Pseudoromiciaprincipis* (Juste, Torrent, Méndez-Rodríguez, Howard, García-Mudarra, Nogueras & Ibáñez, 2023), which is known to be abundant and versatile, using urban, agricultural and forested areas (Rainho et al. 2022). The miniopterid *Miniopterusnewtoni* Bocage, 1889 is endemic to both islands and known to be particularly abundant throughout São Tomé (Rainho et al. 2010).

In addition to the endemics, three insectivorous native bat species are common to both islands. The molossid *Chaerephonpumilus* (Cretzschmar, 1826) is known to forage in open spaces and to roost within cracks in rocks or trees, as well as in buildings (Monadjem et al. 2020). The hipposiderid *Hipposiderosruber* (Noack, 1893) is known to prefer lowland tropical moist forests in relation to drier and less forested areas (Rainho et al. 2010, Rainho et al. 2022). This species displays diurnal activity, although (Russo et al. 2011) indicated that the individuals remain more active during the night. The emballonurid *Taphozousmauritianus* E.Geoffroy, 1818 is found in the less forested northern area of São Tomé and has only been acoustically detected in Príncipe (Rainho et al. 2010). This species is an open-space forager and, in other regions of Africa, this species tends to inhabit savannah woodlands, deliberately avoiding densely forested interiors (Monadjem et al. 2020). São Tomé is additionally occupied by the vespertilionid *Myotistricolor* for which only one roosting site in a sea cave is known (Rainho et al. 2010).

**Figure 1.**
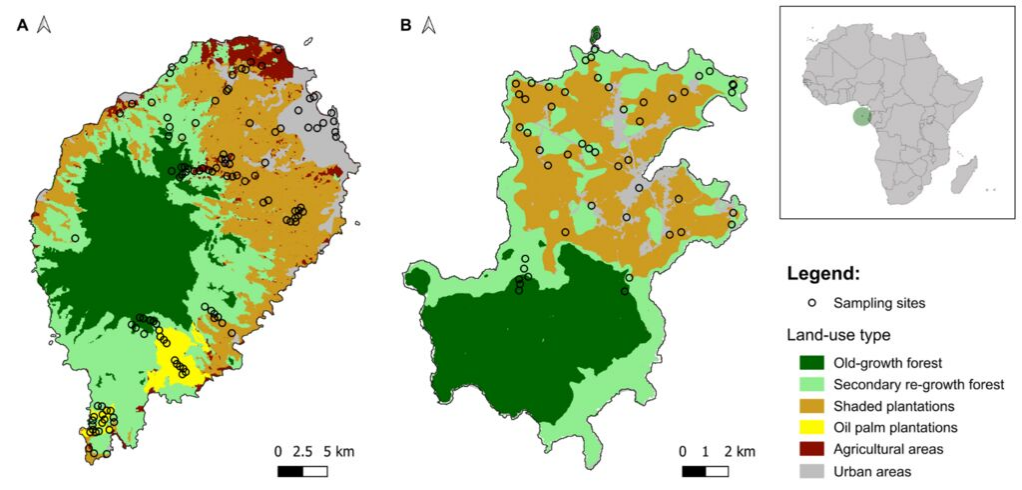
Study area. **A** São Tomé and **B** Príncipe Islands. Sampling sites acoustically surveyed in this study are represented by the dots. Land-use data for São Tomé Island was retrieved from Soares et al. (2020); land-use data for Príncipe Island was retrieved from Soares (2019).

Since human colonisation in the mid-15^th^ century, anthropogenic land-uses have significantly transformed the native landscapes in São Tomé and Príncipe (Lima 2012, Lima et al. 2013, Strauß et al. 2018). These changes resulted in the loss of 74% and 67% of São Tomé and Príncipe native habitats, respectively (Fig. 1). Agriculture first started with the plantation of sugar-cane and subsequently transitioned to cocoa and coffee shade plantations (Jones et al. 1991). Presently, the scenario is further compounded by the extensive spread of oil-palm plantations in the case of São Tomé, which span extensive areas. Although the distribution of species across both islands has been previously assessed (Rainho et al. 2010, Rainho et al. 2022), the understanding of previous and ongoing land-use changes has been hampered by the lack of ground-based data informing how each species uses the different habitat types. In this context, we present a dataset of passive acoustic monitoring data on insectivorous bats collected between August and September 2022 across the main land-use types on the São Tomé and Príncipe Islands.

## Sampling methods

### Study extent

The study was conducted in São Tomé and Príncipe, a democratic state comprising two main islands (São Tomé and Príncipe), as well as several smaller islets. This nation is located in the Gulf of Guinea, off the western equatorial coast of Central Africa (Fig. 1). The islands have a humid tropical climate with four seasons that alternate between rainy and dry periods (Chou et al. 2020). São Tomé has average annual temperatures of 27℃ near the sea level and 21℃ at higher elevations. Príncipe experiences an average annual temperature of 26℃, with minor fluctuations between coastal and mountainous regions (Ministry of Infrastructure, Natural Resources and Environment São Tomé and Príncipe 2015). On both islands, the average annual precipitation ranges from 900 mm in the northeast to around 6,000 mm in the south (Lima and Oliveira 2017). Both islands have rugged mountainous topography, with São Tomé's highest point being Pico de São Tomé at 2024 m above sea level (a.s.l.) and Príncipe's highest point being Pico do Príncipe at 948 m a.s.l.

Tropical forests in São Tomé and Príncipe fall within the ecoregion of São Tomé and Príncipe moist lowland forests. Both islands comprise the Ôbo Natural Park, a significant conservation area established that covers ca. 235 km^2^ in São Tomé (27% of the island area) and 65 km^2^ in Príncipe (46%) (UNEP-WCMC and IUCN 2021). This Park encompasses extensive portions of the islands' old-growth forests and serves as a vital habitat for numerous endemic and threatened species (e.g. the vulnerable dwarf ibis *Bostrychiabocagei*: IUCN (2020); the critically endangered bird *Laniusnewtoni*: Lewis et al. (2018)).

Both islands are occupied by a number of habitat types encompassing a range of human disturbance intensities, including old-growth forests, regrowth forests, shaded plantations, oil-palm plantations (only in São Tomé Island), agricultural areas and urban areas. This dataset includes a compilation of insectivorous bat records obtained across each of these habitats throughout each of the islands (Fig. 1). Old-growth forests comprised the least disturbed areas on the islands, covered by native forest vegetation. While they may have experienced alterations or disturbances in the past, these areas are largely characterised by relatively low levels of known human intervention. Secondary regrowth forests are predominantly composed of introduced species, resulting from the regrowth of vegetation after the removal of pre-existing old-growth forests. Shade plantations represent areas where shade-grown crops, mainly cocoa and coffee, are cultivated beneath the canopy of taller (mostly) exotic trees (Muñoz-Torrent et al. 2022). Oil-palm plantations consist of palm tree monocultures specifically cultivated for organic oil production. Agricultural areas were characterised by landscapes managed by humans for farming activities, cultivating crops like banana trees, cassava, carrots and tomatoes. Urban areas encompass developed regions with human settlements, infrastructure and modified landscapes (Dauby et al. 2022).

### Sampling description

Acoustic surveys targeting insectivorous bats were carried out between August and September 2022, during the dry season. The sampling period was chosen to avoid severe storms typical of the region, preventing rain-induced deterioration of acoustic recordings and ensuring accurate detection of bat activity, which tends to decrease during heavy rain due to reduced insect activity and echolocation interference (Parsons et al. 2003). To ensure data representation for each of the major land-use types present on both islands, sampling sites were tentatively stratified according to the availability of each land-use type. In total, we surveyed 115 sites on the São Tomé Island and 50 sites on the Príncipe Island (Table 1).

Each sampling site was surveyed by deploying one Audio Moth recorder (Hill et al. 2018) programmed to record for one minute every five minutes, for two consecutive days and nights at mid-gain with a 384 kHz sample rate, totalling 240 minutes of active recording per day. Diurnal recording was intended to capture the diurnal activity of *Hipposiderosruber* (*Russo et al. 2011*). Recorders were attached to an available vertical structure, between 1.5 and 2 m above the ground. Spatially contiguous sampling sites were at least 250 m apart from each other to minimise simultaneously recording the same individual (Ferreira et al. 2022). A total of 20 spatially aggregated sampling sites were surveyed at the same time.

Using Kaleidoscope Version 5.4.7 (Wildlife Acoustics, USA), we first split the recordings into five-second WAV files (López‐Baucells et al. 2021). We then filtered the recordings for a frequency range between 8 and 250 kHz and pulses ranging from 2 to 500 milliseconds. Files outside of these parameters were considered ‘noise’ and removed from subsequent analysis. Recordings that contained one bat pass, defined as two or more pulses of a species detected in a five seconds recording (Millon et al. 2015), were then manually classified to the species or genus level. This classification was carried out by examining the following call parameters: frequency of maximum energy (F_maxE_) and minimum energy (F_min_), call duration and shape and inter-pulse interval (Russo and Jones 2002). Information on each of these parameters has been locally obtained for each of the species by Rainho et al. (2022). Echolocation calls were mostly identified at the species level. However, in the case of São Tomé Island, we were not able to distinguish between *C.pumilus* and *C.tomensis* in 16% of the 10,789 *Chaerephon* bat passes due to overlap in the echolocation call parameters of these two species (Rainho et al. 2022). In this case, we refer to *Chaerephon* spp. to include both *C.pumilus* and *C.tomensis* species. Acoustic analysis was the only method of species identification used in this dataset.

## Geographic coverage

### Description

A total of 115 and 50 sampling sites were set up in the São Tomé and Príncipe Islands, respectively. Sampling covered all the predominant land-use types on both islands.

**Coordinates**: Minimum Latitude: 0.029; Maximum Latitude: 1.698; Minimum Longitude: 6.502; Maximum Longitude: 7.457

## Taxonomic coverage

### Description

**Figure 2.**
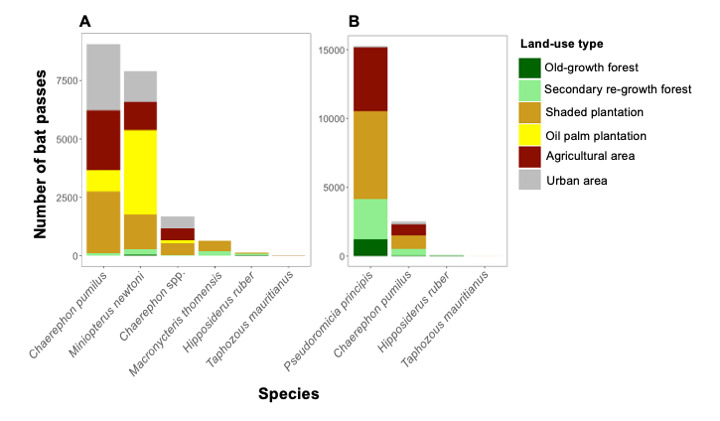
Bat activity (number of bat passes) per species per land-use type in **A** São Tomé and **B** Príncipe Islands.

**Figure 3.**
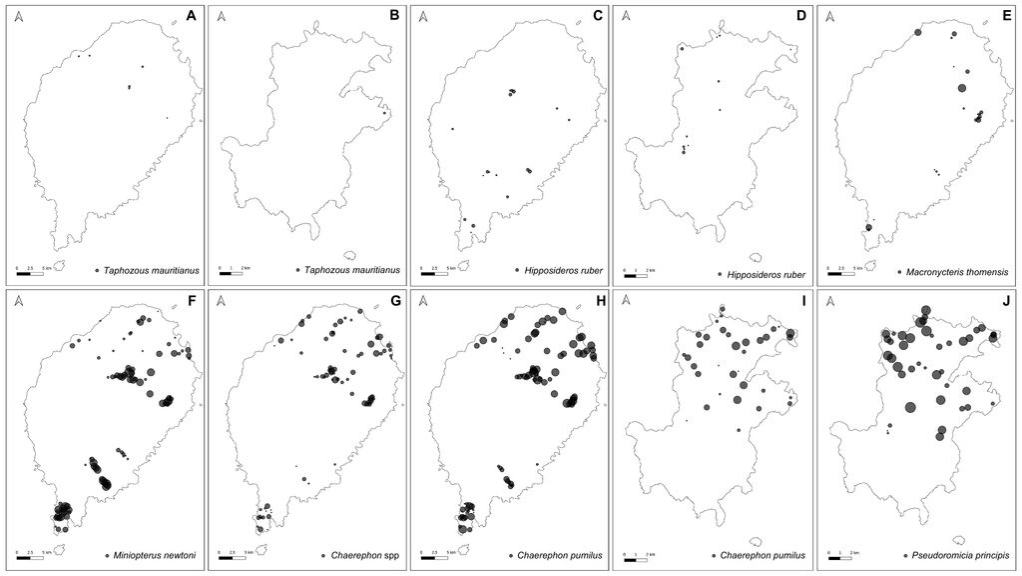
Species-specific maps showing the distribution of each species within São Tomé and Príncipe Islands: *Taphozousmauritianus* in **A** São Tomé and in **B** Príncipe; *Hipposiderosruber* in **C** São Tomé and in **D** Príncipe; **E** *Macronycteristhomensis* in São Tomé; **F** *Miniopterusnewtoni* in São Tomé; **G** *Chaerephon* spp. in São Tomé; *Chaerephonpumilus* in **H** São Tomé and in **I** Príncipe; and **J** *Pseudoromiciaprincipis* in Príncipe. Each point denotes a sampling site where the corresponding species has been recorded. Points are scaled according to the species-specific bat activity recorded (number of bat passes).

São Tomé and Príncipe Islands comprise six and four insectivorous bats, respectively, with the two species, *Chaerephontomensis* and *Macronycteristhomensis*, being island-endemics in São Tomé and *Pseudoromiciaprincipis* being an island-endemic in Príncipe. We recorded a total of 19,437 bat passes across the 115 sites surveyed on São Tomé Island and 17,837 bat passes across the 50 sites surveyed on Príncipe Island (Table 2). *Chaerephonpumilus* exhibited the highest number of bat passes on São Tomé, while *Pseudoromiciaprincipis* showed the highest activity levels on Príncipe Island. Bat activity was higher in anthropogenised habitats compared to natural habitats on both islands (Fig. 2). Notably, *M.newtoni* showed high activity on the oil-palm plantations in the south-south-eastern part of São Tomé Island. In contrast, *Chaerephon* spp. and *Chaerephonpumilus* were most active in the shaded plantations in the northern part of the island. On Príncipe Island, the endemic *P.principis* exhibited the highest activity levels in shaded plantations located in the northern region of the Island. *M.thomensis* showed the lowest activity patterns in São Tomé, while *Taphozousmauritianus* and *Hipposiderosruber* showed the lowest activity patterns on both Islands (Fig. 3).

### Taxa included

**Table:** 

Rank	Scientific Name	
species	Taphozousmauritianus	
species	Hipposiderosruber	
species	Macronycteristhomensis	
species	Miniopterusnewtoni	
genus	Chaerephon spp.	
species	Chaerephonpumilus	
species	Pseudoromiciaprincipis	

## Temporal coverage

**Data range:** 2022-8-01 – 2022-9-12.

## Usage licence

### Usage licence

Creative Commons Public Domain Waiver (CC-Zero)

## Data resources

### Data package title

Insectivorous bat activity dataset across different land-use types in the Islands of São Tomé and Príncipe, Central West Africa

### Resource link

https://doi.org/10.15468/9fgps3

### Number of data sets

1

### Data set 1.

#### Data set name

Insectivorous bat activity dataset across different land-use types in the Islands of São Tomé and Príncipe

#### Data format

Darwin Core Archive format

#### Character set

UTF-8

#### Download URL

http://ipt.gbif.pt/ipt/archive.do?r=event_ipt_stp

#### Description

This dataset provides comprehensive information on the activity of insectivorous bats across different land-use types on the Islands of São Tomé and Príncipe. Acoustic surveys targeting insectivorous bats were carried out in August and September 2022, during the dry season. To ensure data representation for each of the major land-use types present on the Islands, sampling sites were stratified according to the availability of each land-use type. In total, we surveyed 115 sites on São Tomé and 50 sites on Príncipe. Each sampling site was surveyed by deploying one AudioMoth recorder programmed to record for one minute every five minutes, for two consecutive days and nights, totalling 240 minutes of active recording per day. For each bat pass identified, we report the species, geographic coordinates, land-use type, altitude, date and time.

**Data set 1.** 

Column label	Column description
InstitutionID (event core)	An identifier for the institution having custody of the object(s) or information referred to in the record.
institutionCode (event core)	The name (or acronym) in use by the institution having custody of the object(s) or information referred to in the record.
ownerInstitutionCode (event core)	The name (or acronym) in use by the institution having ownership of the object(s) or information referred to in the record.
basisOfRecord (occurrence extension)	The specific nature of the data record.
dynamicProperties (event core)	A list of additional measurements, facts, characteristics or assertions about the record. T: number of trees; HT: average of five highest trees; St: number of stems; L: number of lianas; P: number of palm or coconut trees; C: canopy cover; veg_0: vegetation cover at 0 m; veg_1: vegetation cover at 1 m; veg_2: vegetation cover at 2 m; veg_4: vegetation cover at 4 m; veg_8: vegetation cover at 8 m; veg_16: vegetation cover at 16 m; veg_32vegetation cover at 32 m.
occurrenceID (occurrence extension)	An identifier for the dwc:Occurrence (as opposed to a particular digital record of the dwc:Occurrence).
recordedBy (occurrence extension)	A list (concatenated and separated) of names of people, groups or organisations responsible for recording the original dwc:Occurrence.
individualCount (occurrence extension)	The number of individuals present at the time of the dwc:Occurrence.
organismQuantityType (occurrence extension)	A number or enumeration value for the quantity of dwc:Organisms.
occurrenceStatus (occurrence extension)	A statement about the presence or absence of a dwc:Taxon at a dcterms:Location.
eventID (event core, occurrence extension)	An identifier for the set of information associated with a dwc:Event. In this case, a sampling site.
parentEventID (event core)	An identifier for the broader dwc:Event that groups this and potentially other dwc:Events. In this case, a broader category for a sampling site. STA: São Tomé Agricultural area; STO: São Tomé Palm Oil plantation; STP: São Tomé old growth forest; STR: São Tomé Regrowth forest; STS: São Tomé shaded plantation; STU: São Tomé urban area; PA: Príncipe Agricultural area; PP: Príncipe old growth forest; PR: Príncipe Regrowth forest; PS: Príncipe Shaded plantation; PU: Príncipe Urban area.
eventDate (event core, occurrence extension)	The date-time or interval during which a dwc:Event occurred. For occurrences, this is the date-time when the dwc:Event was recorded.
habitat (event core)	A category or description of the habitat in which the dwc:Event occurred.
samplingProtocol (event core)	The names of, references to, or descriptions of the methods or protocols used during a dwc:Event.
sampleSizeValue (event core)	A numeric value for a measurement of the size (time duration, length, area or volume) of a sample in a sampling dwc:Event.
sampleSizeUnit (event core)	The unit of measurement of the size (time duration, length, area or volume) of a sample in a sampling dwc:Event.
samplingEffort (event core)	The amount of effort expended during a dwc:Event.
continent (event core)	The name of the continent in which the dcterms:Location occurs.
island (event core)	The name of the island on or near which the dcterms:Location occurs.
countryCode (event core)	The standard code for the country in which the dcterms:Location occurs.
decimalLatitude (event core)	The geographic latitude (in decimal degrees, using the spatial reference system given in dwc:geodeticDatum) of the geographic centre of a dcterms:Location.
decimalLongitude (event core)	The geographic longitude (in decimal degrees, using the spatial reference system given in dwc:geodeticDatum) of the geographic centre of a dcterms:Location.
identifiedBy (occurrence extension)	A list (concatenated and separated) of the globally unique identifier for the person, people, groups or organisations responsible for assigning the dwc:Taxon to the subject.
scientificName (occurrence extension)	The full scientific name, with authorship and date information if known. When forming part of a dwc:Identification, this should be the name in lowest level taxonomic rank that can be determined.
kingdom (occurrence extension)	The full scientific name of the kingdom in which the dwc:Taxon is classified.
phylum (occurrence extension)	The full scientific name of the phylum or division in which the dwc:Taxon is classified.
class (occurrence extension)	The full scientific name of the class in which the dwc:Taxon is classified.
order (occurrence extension)	The full scientific name of the order in which the dwc:Taxon is classified.
family (occurrence extension)	The full scientific name of the family in which the dwc:Taxon is classified.
genus (occurrence extension)	The full scientific name of the genus in which the dwc:Taxon is classified.
taxonRank (occurrence extension)	The taxonomic rank of the most specific name in the dwc:scientificName.
eventTime (occurrence extension)	The time or interval during which a dwc:Event occurred.
organismQuantity (occurrence extension)	A number or enumeration value for the quantity of dwc:Organisms.
geodeticDatum (event core)	The ellipsoid, geodetic datum or spatial reference system (SRS), upon which the geographic coordinates given in dwc:decimalLatitude and dwc:decimalLongitude are based.
coordinateUncertaintyInMetres (event core)	The horizontal distance (in metres) from the given dwc:decimalLatitude and dwc:decimalLongitude describing the smallest circle containing the whole of the dcterms:Location.
country (event core)	The name of the country or major administrative unit in which the dcterms:Location occurs.
minimumElevationInMetres (event core)	The lower limit of the range of elevation (altitude, usually above sea level), in metres.
maximumElevationInMetres (event core)	The upper limit of the range of elevation (altitude, usually above sea level), in metres.

## Figures and Tables

**Table 1.** Number of sampling sites where insectivorous bats were surveyed per land-use type in both São Tomé and Príncipe Islands.

**Land-use types**	**São Tomé Island**	**Príncipe Island**
Old-growth forests	13	11
Secondary re-growth forests	17	13
Shaded plantations	29	15
Oil palm plantations	20	-
Agricultural areas	23	9
Urban areas	13	2

**Table 2.** Insectivorous bat list species in São Tomé and Príncipe Islands, Central West Africa. *Chaerephon* spp. corresponds to cases in which *C.pumilus* and *C.tomensis* could not be distinguished due to overlap in these species’ echolocation call parameters.

**Family**	**Scientific name**	**Location**	**Number of bat passes recorded**
Emballonuridae	*Taphozousmauritianus* E.Geoffroy, 1818	São Tomé Island	23
Emballonuridae	*Taphozousmauritianus* E.Geoffroy, 1818	Príncipe Island	7
Hipposideridae	*Hipposiderosruber* (Noack, 1893)	São Tomé Island	129
Hipposideridae	*Hipposiderosruber* (Noack, 1893)	Príncipe Island	48
Hipposideridae	*Macronycteristhomensis* (Bocage, 1891)	São Tomé Island	632
Miniopteridae	*Miniopterusnewtoni* Bocage, 1889	São Tomé Island	7,900
Molossidae	*Chaerephon* Dobson, 1874	São Tomé Island	1,686
Molossidae	*Chaerephonpumilus* (Cretzschmar, 1826)	São Tomé Island	9,067
Molossidae	*Chaerephonpumilus* (Cretzschmar, 1826)	Príncipe Island	2,513
Vespertilionidae	*Pseudoromiciaprincipis* (Juste, Torrent, Méndez-Rodríguez, Howard, García-Mudarra, Nogueras & Ibáñez, 2023)	Príncipe Island	15,269
